# Structural determinants at KCNE4 position 145 govern Kv1.3 channel function

**DOI:** 10.1085/jgp.202513936

**Published:** 2026-05-20

**Authors:** Magalí Colomer-Molera, Daniel Sastre, Antonio Felipe

**Affiliations:** 1 Molecular Physiology Laboratory, Departament de Bioquímica i Biomedicina Molecular, Institut de Biomedicina (IBUB), Universitat de Barcelona, Barcelona, Spain

## Abstract

Kv1.3 channels participate in the activation and proliferation of leukocytes. The KCNE4 regulatory subunit associates with the channel and functions as a negative regulator. KCNE4, via its transmembrane and C-terminal domains, interacts with Kv1.3, impairing forward plasma membrane trafficking, decreasing macroscopic currents, and accelerating slow C-type inactivation of the channel. A negatively charged 145D/E polymorphic variant of KCNE4 has been associated with immune system disorders, such as allergic rhinitis and childhood acute lymphoblastic leukemia. In this work, we investigated the functional effects of these KCNE4 variants on Kv1.3 activity. Both variants similarly impaired forward trafficking of the channel. However, we observed a variant-dependent decrease in Kv1.3 currents with minor kinetic effects. In addition, we explored the effects of different residues at this position and analyzed the importance of the central amino acid of the polymorphism within the anionic (D–D/E–E) triplet. We suggest that the size and charge of the central position of the cluster are crucial for controlling Kv1.3 currents.

## Teaser

This study reveals that a KCNE4 polymorphism differentially regulates Kv1.3 activity. This work highlights how the size and charge of a single residue within an anionic cluster critically shape the function of Kv1.3.

## Introduction

The potassium channel regulatory subunit KCNE family comprises five members of single-spanning transmembrane domain (TMD) (type I) proteins (McCrossan and Abbott, 2004) that associate with different voltage-gated potassium (Kv) channels (Hille, 2001). KCNE regulation of Kv channels increases their functional diversity, enabling their participation in controlling gating kinetics, conductance, inactivation kinetics, plasma membrane trafficking, voltage dependence, and drug sensitivity (McCrossan and Abbott, 2004; Kanda and Abbott, 2012). The association of specific KCNE regulatory subunits with specific Kv channels is mandatory to recapitulate native currents, such as the cardiac I_Ks_ current generated by the association between KCNE1 and Kv7.1 (Sanguinetti et al., 1996; Lundquist et al., 2005). Evidence suggests that mutations in some KCNE members are strongly related to several human channelopathies (Sesti and Goldstein, 1998).

The Kv1.3 channel is essential to leukocyte physiology. KCNE4 regulates Kv1.3 (Grunnet et al., 2003). KCNE4 is the largest member of the KCNE family and has the longest C-terminal (Ct) domain. Both proteins are expressed in tissues, such as the immune system (Grissmer et al., 1990; Cahalan and Chandy, 2009; Vallejo-Gracia et al., 2021), brain, and muscle (Veh et al., 1995; Teng et al., 2003; Xu et al., 2004).

The main effect of KCNE4 on Kv1.3 activity is a substantial reduction in the current of the channel (Grunnet et al., 2003; Sole et al., 2009; Grunnet et al., 2020; Vallejo-Gracia et al., 2021; Sastre et al., 2024b). This inhibition is linked to KCNE4-dependent impairment of Kv1.3 plasma membrane targeting (Sole et al., 2009). Only KCNE4, out of the five KCNE family members, is able to modulate Kv1.3 trafficking (Sole et al., 2009; Sastre et al., 2024b). In addition to current amplitude, KCNE4 also regulates the characteristic slow C-type inactivation of the channel (Attali et al., 1992). KCNE4 accelerates this inactivation in a stoichiometry-independent mechanism (Sole et al., 2020) via interactions with the TMD and Ct domains of the regulatory protein (Sastre et al., 2024b).

Kv1.3 is important in leukocyte physiology for controlling T lymphocyte activation (Nguyen et al., 1996), proliferation (Conforti et al., 2003), and cytokine production (Hu et al., 2007). In this scenario, altered Kv1.3 activity is linked to autoimmune diseases such as multiple sclerosis (Beeton et al., 2001), rheumatoid arthritis (Beeton et al., 2006), or psoriasis (Kundu-Raychaudhuri et al., 2014). Consequently, Kv1.3 is of great therapeutic interest in the treatment of these disorders (Navarro-Perez et al., 2024). On the other hand, the role of KCNE4 in the pathogenesis of the immune system has been less explored. Kv1.3-associated T-cell physiological events are affected by changes in KCNE4 abundance (Vallejo-Gracia et al., 2021). The physiological role of KCNE4 is just emerging, and to completely understand the function of this ancillary subunit, further studies are needed.

In this work, we focused on a specific human KCNE4 polymorphism. The amino acid variation, which is located in the intracellular Ct domain of the protein, is correlated with immune system alterations such as childhood acute lymphoblastic leukemia (ALL) (Trevino et al., 2009) and allergic rhinitis (Freidin et al., 2013). This single-nucleotide polymorphism (SNP) affects a distal 145 position and involves an aspartic acid (Asp) residue (wild type [WT]) and a glutamic acid (Glu) in the polymorphic variant. Although both amino acids are similar with negatively charged side chains, we detected a variant-dependent reduction in Kv1.3 current. We analyzed how this KCNE4 minimal missense mutation affects Kv1.3 behavior. The effect of this SNP on Kv1.3, which is crucial in the immune system physiology, should be evaluated further in the context of the human KCNE4-related pathologies.

## Materials and methods

### Constructs

Human KCNE4 (short isoform) in pXOOM and human Kv1.3 in pRcCMV were generous gifts from J. Barhanin (Université Côte d’Azur, Nice, France) and F. Bezanilla (University of Chicago, Chicago, IL, USA), respectively. KCNE4 was subcloned and inserted into pECFP-N1 (Clontech Laboratories). Kv1.3 was subcloned and inserted into pEYFP-C1 (Clontech Laboratories) and pcDNA3. pDsRed-tagged pleckstrin homology domain of Akt, which is used as a transfectable membrane marker, was a gift from F. Viana (Universidad Miguel Hernández, Elche, Spain). KCNE4 mutants were generated using an Agilent QuikChange II site–directed mutagenesis kit and confirmed by Sanger sequencing.

### Cell culture

Human embryonic kidney 293 (HEK293) cells were cultured in Dulbecco’s modified Eagle’s medium supplemented with 10% fetal bovine serum, 10,000 U/ml penicillin, and 100 μg/ml streptomycin (Thermo Fisher Scientific).

For electrophysiological experiments, cells were seeded in 35-mm dishes and transfected with lipotransfectin (Attendbio Research) according to the supplier’s instructions with 150 ng (Kv1.3) and 450 ng (KCNE4) of DNA. In the immunoprecipitation studies, HEK293 cells in a 100-mm dish were transfected with 4 μg of each DNA. For confocal imaging, cells were seeded on coverslips coated with poly-D-lysine and transfected with 750 ng of each DNA. All the experiments were performed 24–36 h after transfection.

### Protein extraction, coimmunoprecipitation, and western blotting

Cells were washed twice in cold phosphate-buffered saline (PBS) and scraped in lysis buffer (1% Triton X-100, 10% glycerol, 50 mM HEPES, and 150 mM NaCl, pH 7.2) supplemented with the following protease inhibitors: 1 μg/ml aprotinin, 1 μg/ml leupeptin, 1 μg/ml pepstatin, and 1 mM PMSF (Sigma-Aldrich). The homogenates were subsequently centrifuged at 15,200 × *g* for 10 min, after which the supernatants were collected. The protein concentration was determined using Bio-Rad Bradford Assay Kit (Bio-Rad).

Up to 2 mg of protein was precleared with 50 μl of Protein A Sepharose beads (GE Healthcare) in 500 μl of washing buffer (150 mM NaCl, 50 mM HEPES, and 1% Triton X-100, pH 7.4) supplemented with protease inhibitors at 4°C with gentle mixing for 1 h. The precleared samples were incubated in Micro Bio-Spin Chromatography Column (Bio-Rad) for 2 h at room temperature (RT) with gentle mixing. The columns had been previously incubated for 1 h at RT with 50 μl of Protein A Sepharose beads crosslinked to 2.5 μl of anti-GFP antibody (GenScript) with 2.6 mg of dimethyl pimelimidate (Santa Cruz Biotechnology) for 30 min at RT with gentle mixing and washed with Tris-buffered saline. The columns were washed four times before elution with 0.2 M glycine (pH 2.5).

The samples were loaded on SDS–PAGE gels for protein electrophoresis and transferred to PVDF membranes (MilliporeSigma) and blocked in PBS with 0.05% Tween-20 and 5% dry milk for 1 h. The membranes were immunoblotted with antibodies against Kv1.3 (1/200; L23/27; NeuroMab) or GFP (1/200; 11814460001; Roche) overnight at 4°C or with β-actin (1/10,000; A5441; Sigma-Aldrich) at RT for 45 min. Blot images were obtained after the membranes were incubated with horseradish peroxidase anti-mouse secondary antibodies (1721011; Bio-Rad).

### Confocal microscopy and image analysis

The seeded coverslips were washed twice with prewarmed (37°C) PBS without K^+^ and fixed for 10 min with prewarmed 4% paraformaldehyde (Sigma-Aldrich). Coverslips were mounted on microscope slides (Acefesa) with Mowiol-DABCO (Merck Millipore) and dried at RT for at least 3 days before imaging.

Images were obtained on a ZEISS LSM 880 laser scanning confocal spectral microscope (Carl Zeiss AG) using a 63× oil-immersion objective lens with a numerical aperture of 1.4. Image analysis was performed using ImageJ (National Institutes of Health). A pixel-by-pixel triple-colocalization analysis was performed using the plugin JACoP as previously described (Sastre et al., 2019), and Manders’ overlap coefficients (MOCs) were calculated.

### Flow cytometry

The quantification of the fraction of protein at the plasma membrane was based on a previously published method (Bourdin et al., 2016). Nonpermeabilized HEK293 cells transfected with Kv1.3-hemagglutinin (HA) in the presence or absence of KCNE4-CFP constructs and resuspended in PBS were incubated with an anti-HA antibody (H6908; Sigma-Aldrich) conjugated with Cy5 at a 1:1,000 dilution at RT for 45 min. After washing, the presence of Kv1.3-HA at the plasma membrane was quantified as the Cy5 intensity in a BD FACSAria cell sorter. Cell viability was analyzed with propidium iodide staining. For data visualization, OMIQ (Dotmatics) software was used.

### Electrophysiology

Transfected HEK293 cells were trypsinized and replated on a perfusion chamber. After 15 min, the cells were extensively washed with extracellular solution containing (in mM) 145 NaCl, 4 KCl, 1 MgCl_2_, 1.8 CaCl_2_, 10 HEPES-Na, and 10 glucose at a pH of 7.4 adjusted with NaOH. Borosilicate electrodes were fabricated from glass capillaries (1.2 OD × 0.94 × 100 L mm) (Harvard Apparatus) with a P-97 puller (Sutter Instruments) and fire-polished with an MF-830 microforge (Narishige) to achieve an average resistance of 2–4 MΩ. The intracellular pipette filling solution contained (in mM) 80 AspK, 42 KCl, 10 KH_2_PO_4_, 5 EGTA-K, 5 HEPES-K, 3 phosphocreatine, and 3 ATP-Mg adjusted to pH 7.2 with KOH. Cells with comparable fluorescence intensities for YFP (Kv1.3) and CFP (KCNE4) were selected for patch clamp recordings to ensure similar levels of transfection across experiments. After gigaseal formation, the cells were clamped at a holding potential of −80 mV. The K^+^ currents were recorded at RT (22–25°C) using the whole-cell configuration of the patch clamp technique with an EPC-10 amplifier (HEKA Elektronik GmbH), allowing 45 s for complete recovery of Kv1.3 from inactivation between pulses (Sastre et al., 2024a). Currents were sampled at 10 kHz and filtered at 2 kHz. Series resistance was compensated automatically (from 30 to 75%) using PatchMaster software when it was higher than 5 MΩ. For IV curves, cells were stimulated with 250-ms square pulses ranging from +60 to −80 mV in 10-mV steps to elicit voltage-gated currents with tail currents measured at −40 mV. The peak current was normalized by the cell capacitance to obtain the current density. The activation, inactivation, and deactivation processes were fitted to a monoexponential equation as follows:

y=A0+A1exp(−t/τ),

where τ represents the time constant, A0 the baseline value, and A1 the amplitude. Conductance–voltage curves were fitted using a Boltzmann equation:

$$y=1/\left\{1+\exp\left[\left(-\left(V-V_{50}\right)/s\right)\right]\right\},$$

where s represents the slope factor, V represents the membrane potential, V_50_ represents the voltage at which 50% of the channels are open, and y represents the relative current. Square pulses at +60 mV for 5 s were used for C-type inactivation analysis, from which the percentage of inactivation at the end of a pulse and inactivation constant (τ_inactivation_) were obtained. A train of 15 depolarizing pulses at +60 mV was used for cumulative inactivation studies. The current of each individual pulse was normalized against the initial current and plotted to fit an exponential decay curve to calculate the decay constant of inactivation. Steady-state inactivation was measured by applying a +40-mV test pulse after a 2-s prepulse ranging from −40 mV to +60 mV in 10-mV steps. The currents were measured, plotted against the voltage, and fitted to a Boltzmann equation from which V_50_ and slope (s) values were calculated. For inactivation kinetic analysis, only currents <10 nA were considered to avoid introducing voltage error.

PatchMaster and FitMaster v2x90.5 (HEKA Elektronik GmbH) software packages were used for recording and analyzing the currents, respectively.

### Statistical analysis

The results are expressed as individual values with the mean ± SE. Normality of the residuals was assessed using the Shapiro–Wilk test and the visual examination of the QQ plots. Homoskedasticity was addressed following the Brown–Forsythe test. Data from conditions following a normal distribution were analyzed with a one-way ANOVA and Tukey’s post hoc test. Welch’s ANOVA test with unpaired *t* test with Welch’s correction or Kruskal–Wallis and Dunn’s multiple comparisons test were used for experiments with significant P values for the normality and homoskedasticity tests. For coimmunoprecipitation (coIP) experiments, a repeated-measures one-way ANOVA was performed. For flow cytometry experiments, significance was calculated with a Brown–Forsythe ANOVA test. All statistical analyses were performed with GraphPad 8. Differences were considered significant when P < 0.05. A complete summary of statistical analysis is depicted in Table S1.

### Online supplemental material

**Figure S1.**
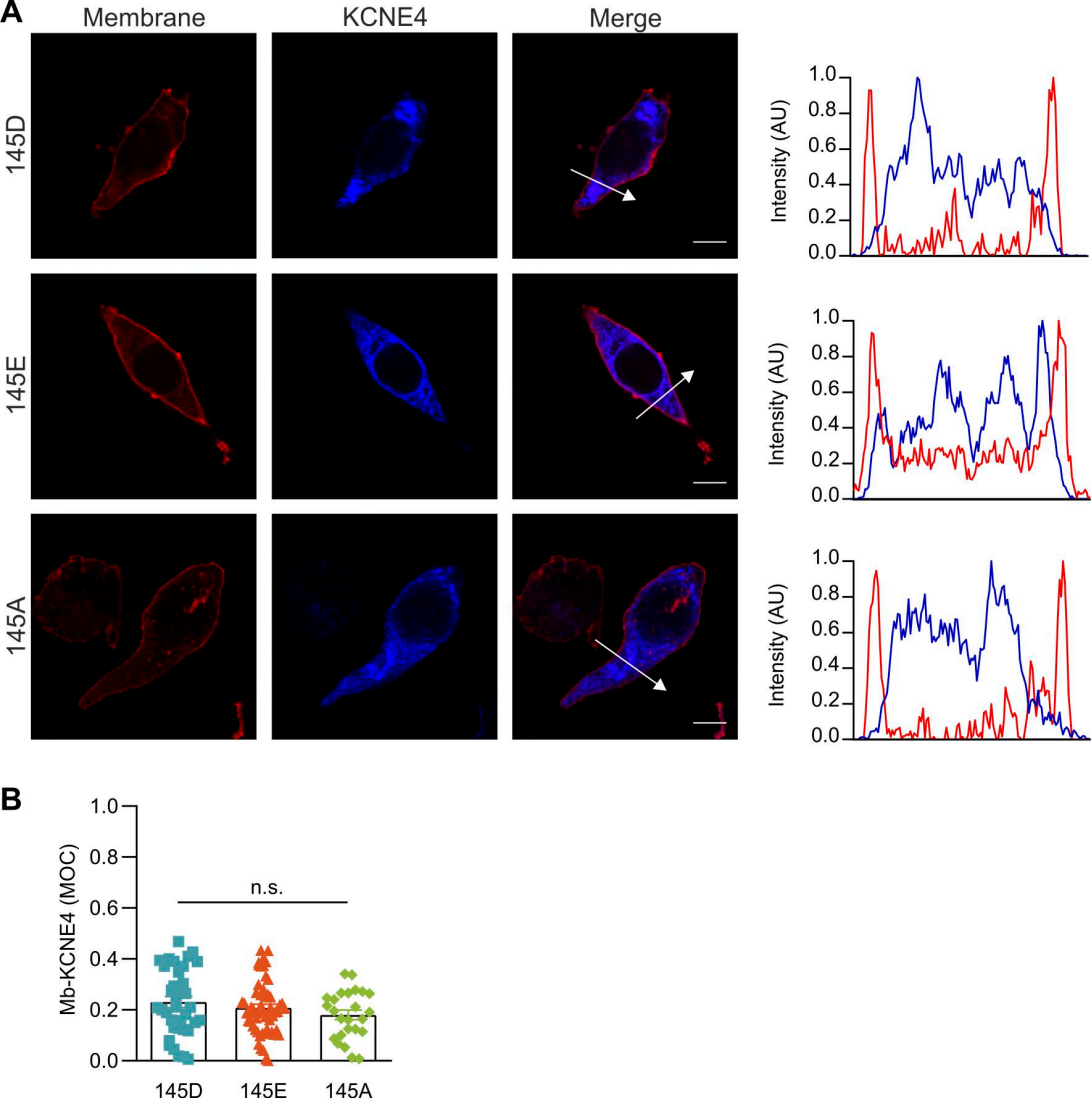
**Plasma membrane trafficking of the KCNE4 variants. (A)** Representative confocal images of HEK293 cells transfected with KCNE4-CFP 145D, 145E, and 145A (blue) and a DsRed membrane marker (red). The panels on the right show the respective pixel-by-pixel profiles indicated by the white arrows in the merged panels. Blue, KCNE4 variants; red, membrane marker. **(B)** MOC for KCNE4 overlapping with the membrane marker (Mb). The individual values and the mean ± SE were plotted for KCNE4 145D (37 cells), 145E (55 cells), and 145A (25 cells). Statistical analysis: one-way ANOVA. P: 0.225, *n*.s., not significant.

**Figure S2.**
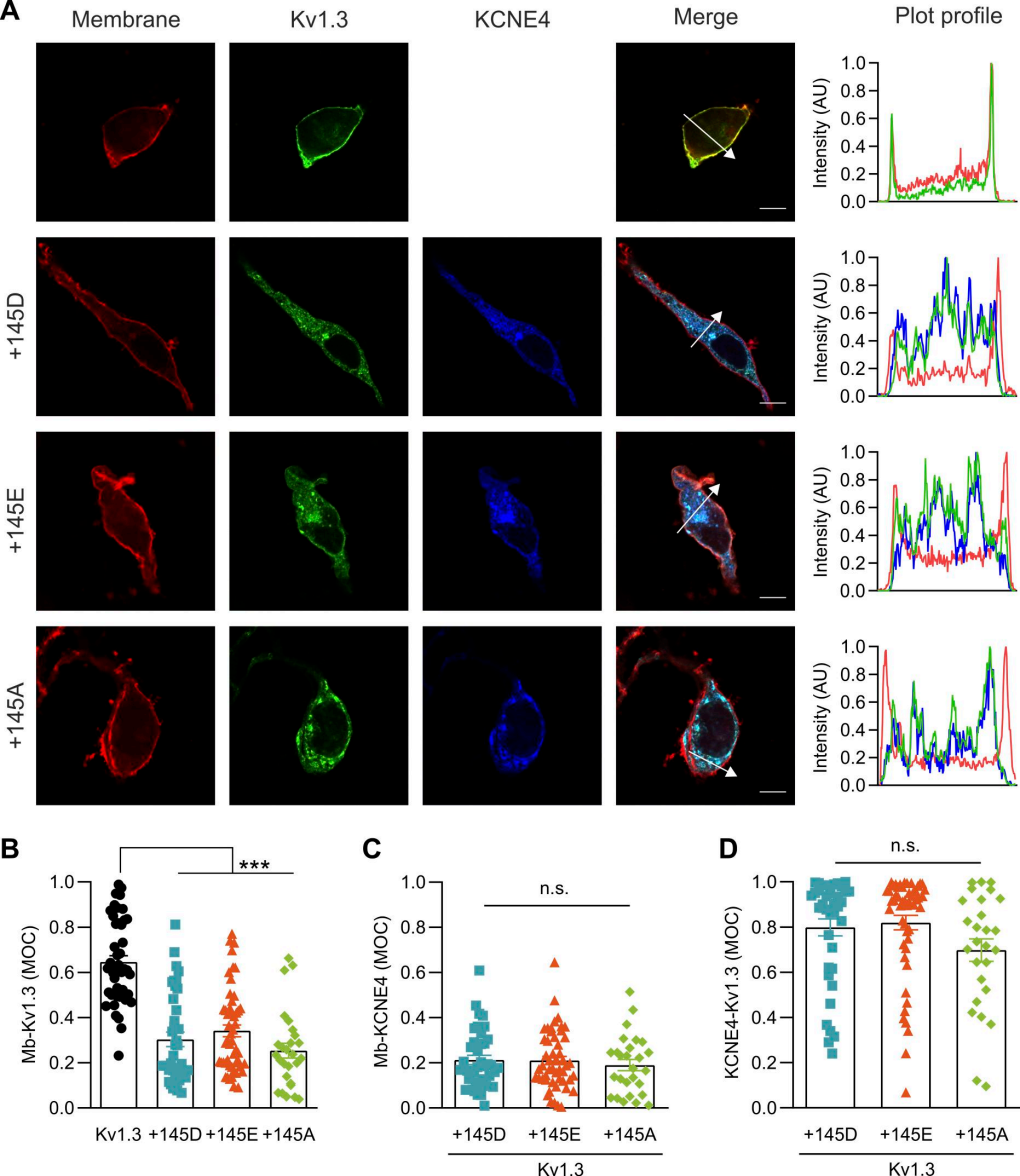
**Both KCNE4 (145D and 145E) variants similarly impair the plasma membrane targeting of Kv1.3.** Representative confocal microscopy images of a triple-colocalization experiment of Kv1.3, KCNE4, and DsRed membrane marker. **(A)** Representative images of HEK293 cells transfected with a membrane marker (red), Kv1.3-YFP (green), and KCNE4-CFP variants (blue). In the right panels, the plot profiles correspond to the sections indicated with white arrows in the merged panels. Scale bar: 10 µm. **(B)** MOC of Kv1.3 at the plasma membrane (Mb) in the absence (Kv1.3 alone, 45 cells) or in the presence of KCNE4 variants (+145D, 39 cells; +145E, 51 cells; or +145A, 27 cells). P: <0.0001. **(C)** MOC for KCNE4 present at the plasma membrane (Mb) in the presence of Kv1.3. P: 0.764. **(D)** MOC for Kv1.3 colocalization with KCNE4. P: 0.093. Individual values and mean ± SE were plotted. Statistical analysis: Kruskal–Wallis with Dunn’s multiple comparisons test. *n*.s., not significant. ***P < 0.001. Black circles, Kv1.3 alone; blue squares, +145D; orange triangles, +145E; and green diamonds, +145A.

**Figure S3.**
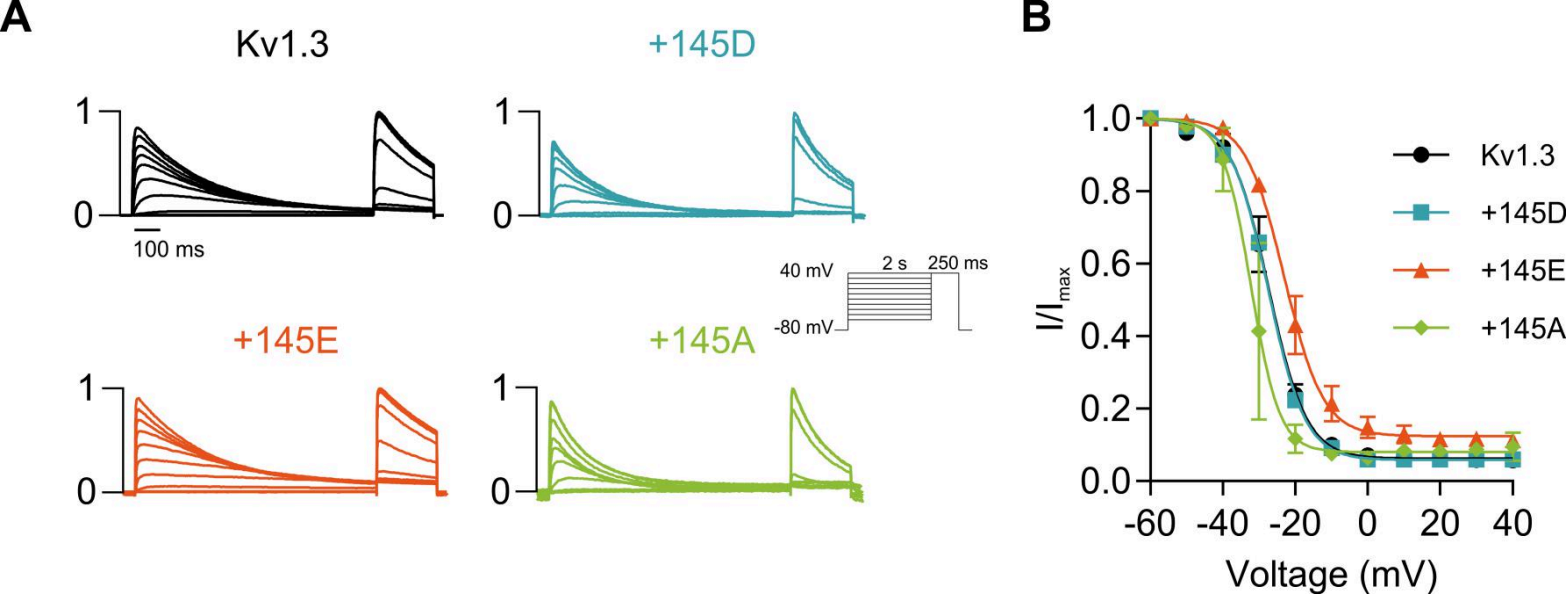
**Steady-state inactivation of Kv1.3 in the presence of KCNE4 variants.** HEK293 cells were transfected with Kv1.3 in the absence or presence of different KCNE4 variants (+145D, +145E, +145A). **(A)** Representative two-step currents analyzing the steady-state inactivation of Kv1.3. The cells were held at −80 mV. Currents were elicited by a 2-s depolarizing prepulse from −60 mV to +40 mV followed by a 250-ms test pulse at +40 mV to measure the voltage dependence of inactivation. **(B)** Relativized peak currents of the test pulse were fitted to a Boltzmann equation. Black circles, Kv1.3 alone; blue squares, +145D; orange triangles, +145E; and green diamonds, +145A. The values represent the mean ± SE of 2–3 cells.

Fig. S1 shows confocal images of plasma membrane trafficking of the KCNE4 variants. Fig. S2 presents confocal microscopy images demonstrating that both KCNE4 (145D and 145E) variants similarly impair the plasma membrane targeting of Kv1.3. Fig. S3 analyzes the steady-state inactivation of Kv1.3 in the presence of KCNE4 variants. Table S1 summarizes the statistical analyses performed, including P values for the Shapiro–Wilk normality test, Brown–Forsythe test for homoskedasticity, and the statistical tests used to compare differences between conditions. Table S2 shows the voltage-dependent activation constant and τ_inactivation_ of Kv1.3 in the presence of KCNE4 variants. Table S3 presents the time constants for the activation and deactivation of Kv1.3 with the polymorphic KCNE4 variants. Table S4 shows the percentage of inactivation and τ of Kv1.3 current inactivation, as well as decay constants from a train of 15 depolarizing pulses.

## Results

### The 145D/E polymorphism of KCNE4 differentially affects the current density of Kv1.3

**Figure 1.**
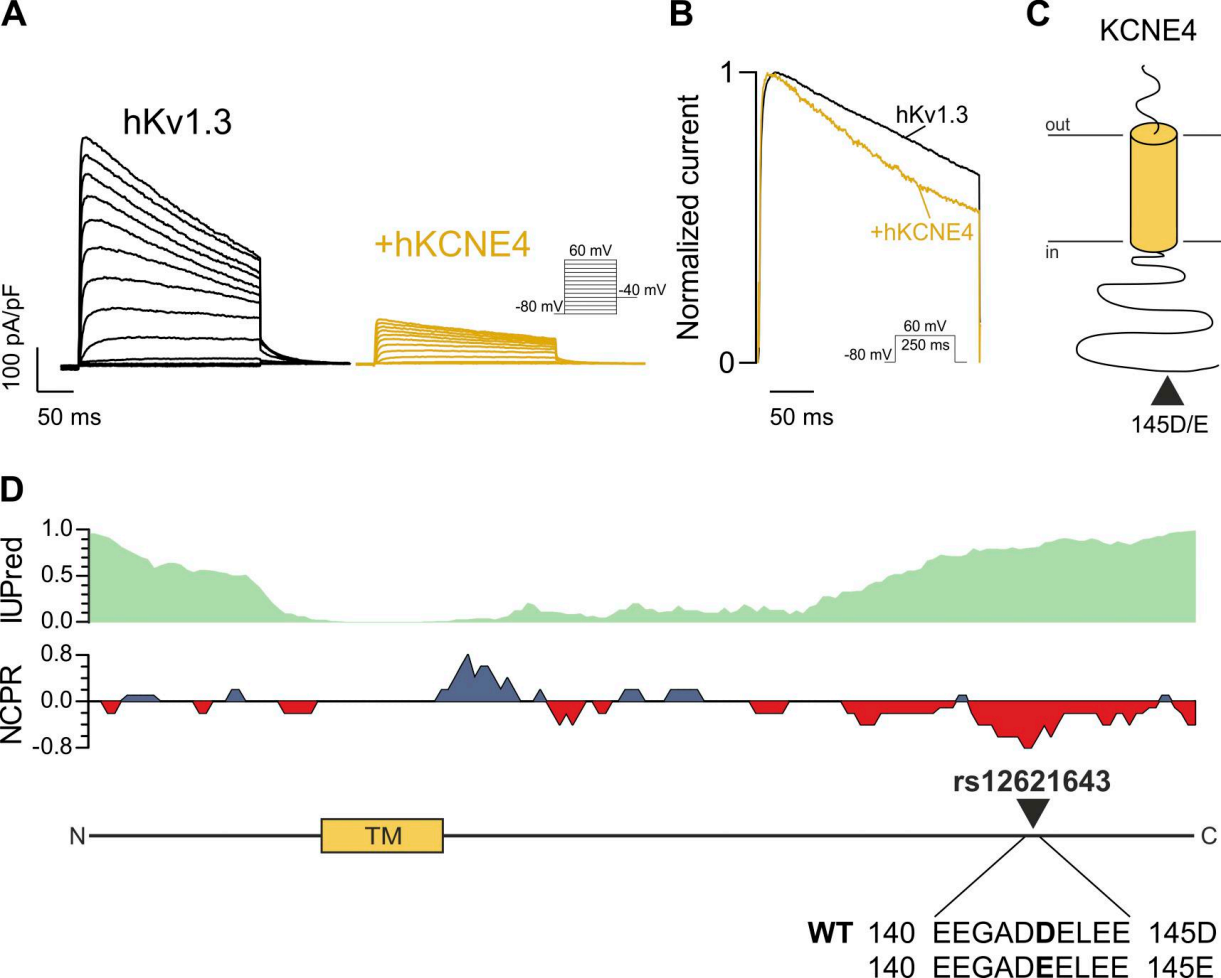
**Human KCNE4 145D/E polymorphism. (A)** Presence of human KCNE4 reduces Kv1.3 currents. HEK293 cells were transfected with hKv1.3 without or with (+) hKCNE4. Currents were elicited with 250-ms-long pulses from −80 mV to +60 mV in 10-mV steps. **(B)** Normalized peak current density of hKv1.3 in the absence or presence (+) of hKCNE4 at +60 mV. KCNE4 accelerates slow C-type inactivation of Kv1.3. **(C)** Schematic cartoon of the KCNE4 regulatory subunit with the arrowhead indicating the distal location of the rs12621643 (145D/E) polymorphism. **(D)** KCNE4 with predicted sequence disorder (top), residue charge (middle), and TMD (orange box) and 145 polymorphism location sequence (bottom). Blue represents basic (positively charged) residues, and red represents acidic (negatively charged) residues.

**Figure 2.**
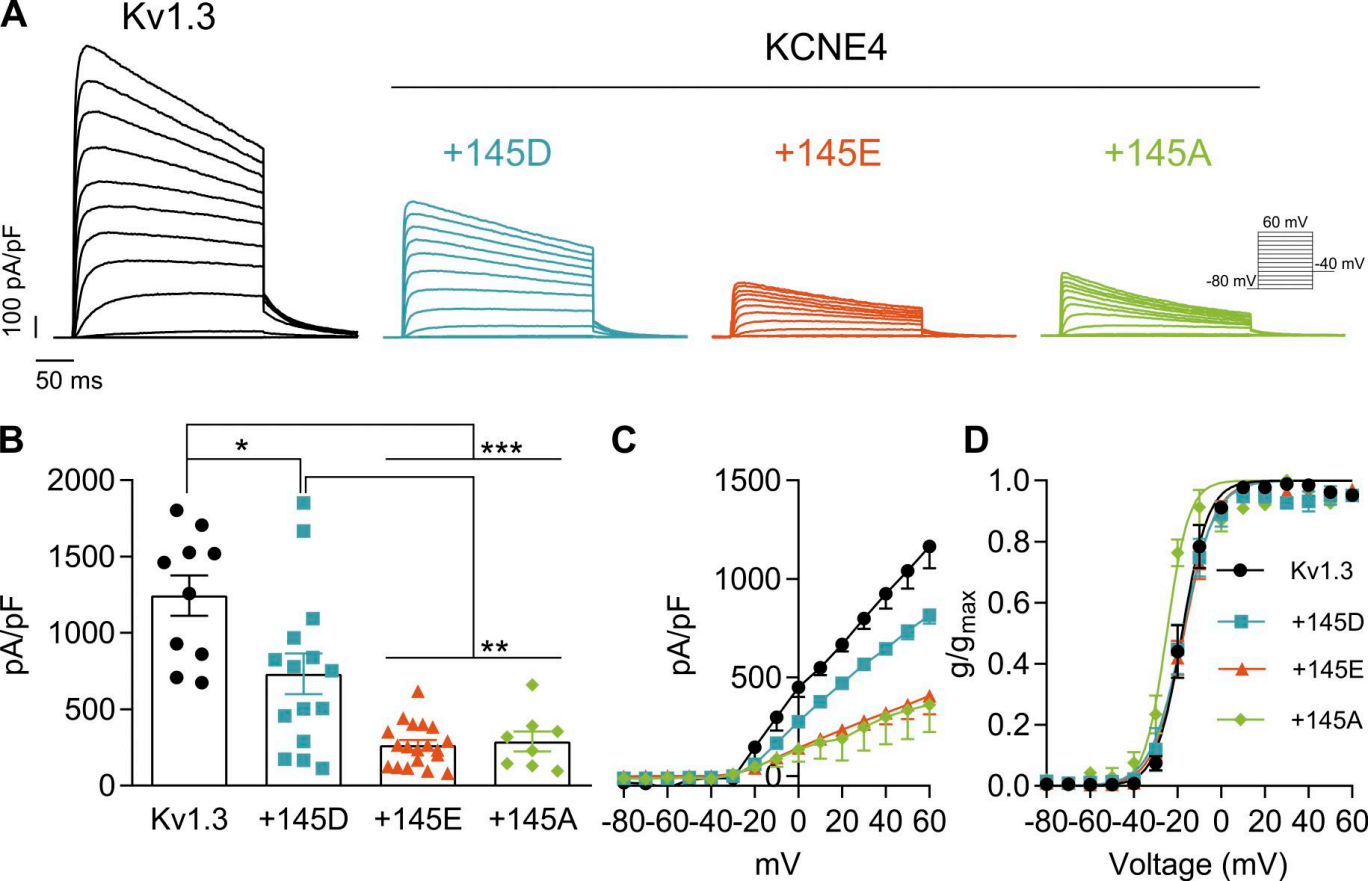
**KCNE4 145D/E polymorphism differentially decreases Kv1.3 currents.** HEK293 cells were transfected with Kv1.3 in the absence or presence (+) of different variants of KCNE4 (+145D, +145E, and +145A). **(A)** Representative currents of Kv1.3. Currents were elicited by 250-ms-long pulses from −80 mV to +60 mV in 10-mV steps and tail currents measured at −40 mV. **(B)** Peak current density was measured at +60 mV for Kv1.3 either without (1,245 ± 133 pA/pF, *n* = 10) or with different KCNE4 mutants (+145D [732 ± 133 pA/pF, *n* = 13], +145E [266 ± 32 pA/pF, *n* = 19], and +145A [290 ± 66 pA/pF, *n* = 8]). Statistical analysis: Welch’s ANOVA test (P < 0.0001) and unpaired *t* test with Welch’s correction. Specific P values were as follows: *P < 0.05 (0.0123, Kv1.3 vs. +145D); **P < 0.01 (0.0038, +145D vs. +145E; 0.0077, +145D vs. +145A); ***P < 0.001 (<0.0001, Kv1.3 vs. +145E; <0.0001, Kv1.3 vs. +145A). **(C)** IV relationship for Kv1.3 currents in the absence or presence of different KCNE4 variants. **(D)** GV plot of Kv1.3 tail currents measured at −40 mV. Black circles, Kv1.3 alone; blue squares, +145D; orange triangles, +145E; and green diamonds, +145A. The values represent the mean ± SE of 5–8 independent cells for each condition in 5–8 experiments. Recordings were obtained from multiple independent transfections as follows: Kv1.3 (8), +145D (8), +145E (11), and +145A (6). In each experiment, two different random conditions were recorded. GV plot, conductance–voltage plot. n, number of cells.

As previously reported (Grunnet et al., 2003; Sole et al., 2009), the presence of KCNE4 decreased Kv1.3 currents (Fig. 1 A) and accelerated slow C-type inactivation of the channel (Fig. 1 B). KCNE4 presents a nonsynonymous SNP in the Ct domain of the protein (rs12621643) resulting in a missense mutation from Asp (D) to Glu (E) at position 145 on hKCNE4. Although D represents the WT variant, E is the most prevalent residue at this position (Table 1). This SNP is localized in an intrinsically disordered region with a high density of negatively charged amino acids (Fig. 1, C and D). Different reports have correlated this polymorphism with the immune system such as childhood ALL (Trevino et al., 2009) and allergic rhinitis (Freidin et al., 2013), as well as cardiovascular disorders (Table 2). We first aimed to analyze how this KCNE4 minimal missense mutation affects Kv1.3 behavior. For that reason, we analyzed the effect of the KCNE4 145D/E polymorphism on Kv1.3 currents using the whole-cell patch clamp technique. Because the natural variants 145D and 145E preserve the negative charge, we also generated a neutral mutation to alanine (145A). Surprisingly, while KCNE4 145D decreased the Kv1.3 peak current density twofold, both 145E and 145A were responsible for the fourfold reduction in the current (Fig. 2, A–C). This difference in current density was not a result of changes in the voltage dependence of activation (Fig. 2 D and Table S2). Furthermore, the presence of either polymorphic KCNE4 variant did not affect the activation or deactivation kinetics of Kv1.3 (Table S3). KCNE4 variants accelerated slow C-type inactivation and decreased the remaining current at the end of a 250-ms-long pulse to a similar degree (Fig. 2 A and Table S4), as previously reported (Sastre et al., 2024b).

**Table 1.** Population frequency of the KCNE4 rs12621643 variant

Population	G (145E) allele frequency	Total allele count	145 EE homozygous
Admixed American	0.7536	60,010	17,228
African/African American	0.5216	74,790	10,174
Amish	0.6421	908	189
Ashkenazi Jewish	0.5549	29,608	4,549
East Asian	0.6718	44,828	10,182
European (Finnish)	0.7851	63,836	19,696
European (non-Finnish)	0.7073	1,179,854	295,215
Middle Eastern	0.5358	6,060	901
South Asian	0.7953	91,062	28,952
Others	0.6859	62,500	14,782
XX	0.7003	812,118	200,499
XY	0.7062	801,338	201,369
Total	0.7032	1,613,456	401,868

Alternative allele frequency, total allele count, and number of individuals homozygous for the alternative allele across populations were obtained from the Genome Aggregation Database (gnomAD, exomes + genomes, v4.1.0[DS2.1]) (Chen et al., 2024). Populations follow gnomAD ancestry group definitions.

**Table 2.** List of diseases and their correlation with the KCNE4 polymorphism

Disease correlation	Variant	References
Atrial fibrillation	145E	(Miao et al., 2017) (Qin et al., 2022)
	145D	(Zeng et al., 2005)
	No correlation	(Sinner et al., 2011) (Abunimer et al., 2014)
Allergic rhinitis	145E	(Freidin et al., 2013)
Childhood ALL	145D	(Trevino et al., 2009)

### Kv1.3 plasma membrane trafficking is impaired by all KCNE4 variants

**Figure 3.**
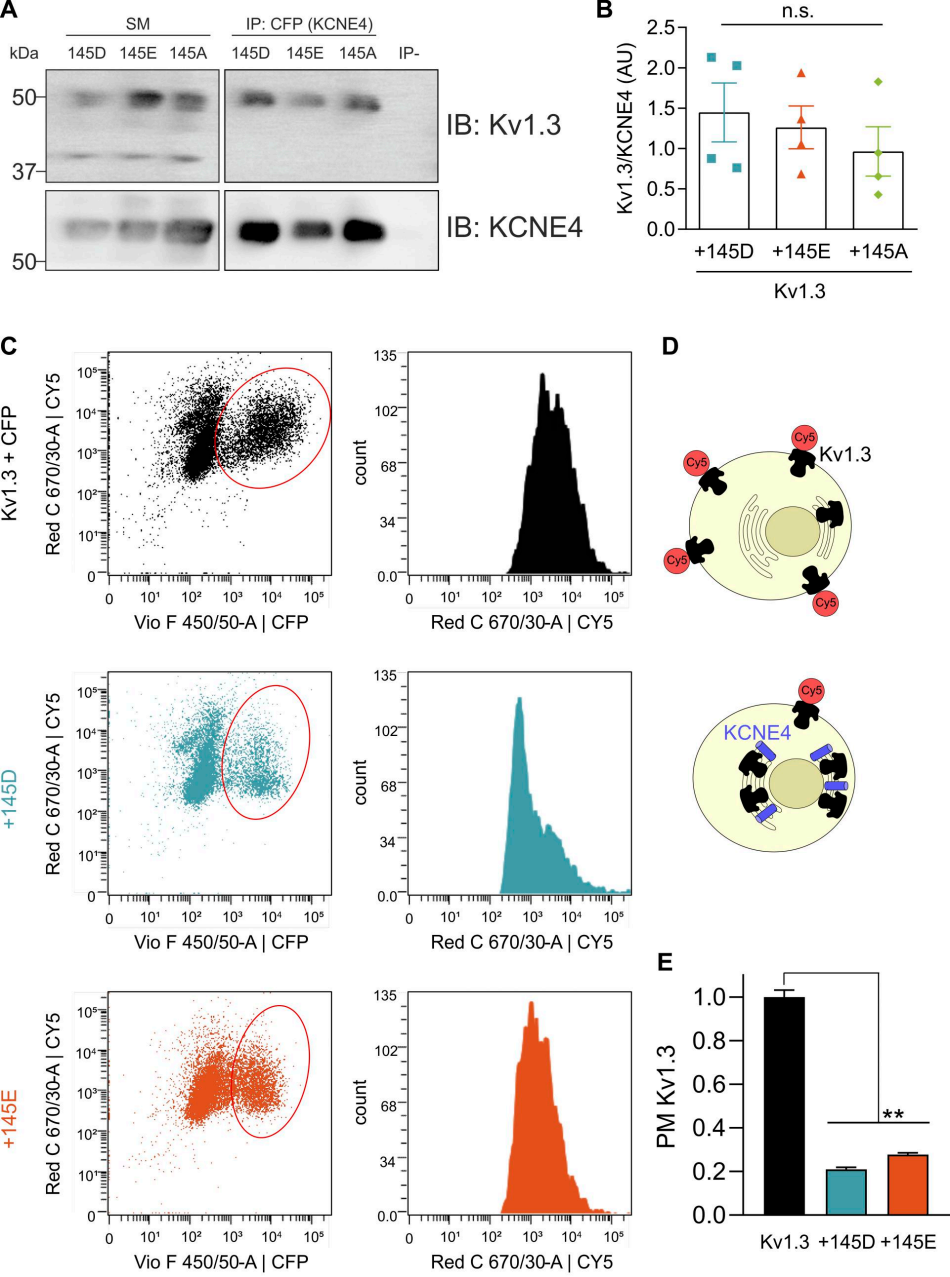
**Association and plasma membrane targeting of Kv1.3 in the presence of KCNE4 are not affected by the 145D/E polymorphism.** HEK293 cells were transfected with Kv1.3 and different KCNE4 variants (145D, 145E, and 145A). **(A)** Representative coIP experiments. Total lysates were IP against CFP (KCNE4) and IB against Kv1.3 and KCNE4 (CFP). SM, starting material; IP-, absence of antibody. **(B)** Quantification of Kv1.3-KCNE4 coIP results from four independent experiments. The values represent the mean ± SE. Statistical analysis: RM one-way ANOVA. **(C–E)** HEK293 cells were transfected with Kv1.3-HA with or without KCNE4-CFP variants. Nonpermeabilized cells were incubated with an anti-HA antibody and a secondary antibody conjugated to Cy5. The Cy5 intensity was measured by flow cytometry. **(C)** Plot of HEK293 cells expressing Kv1.3-HA and CFP as a negative control (top), Kv1.3 + KCNE4 145D (center), and Kv1.3 + KCNE4 145E (bottom) with Cy5 (y axis) and CFP (x axis) fluorescence intensity for each combination (left). On the right, histogram for Cy5 intensity of the CFP-positive cells for each condition (circled in red in the left). **(D)** Schematic representation of cells expressing Kv1.3-HA (black) without (on the left) or with KCNE4 (blue barrel, on the right) with Cy5 (red circle) conjugated to Kv1.3 at the plasma membrane. **(E)** Quantification of plasma membrane Kv1.3 (PM Kv1.3) normalized to Cy5 intensity. The values represent the mean ± SE of at least 1,500 cells for each condition. Statistical analysis: Brown–Forsythe ANOVA test. Specific P values were as follows: **P < 0.01 (0.0016 vs. +145D; 0.0017 vs. +145E). IP, immunoprecipitated; IB, immunoblotted. Source data are available for this figure: SourceData F3.

KCNE4 association impairs the forward plasma membrane trafficking of Kv1.3 (Sole et al., 2009; Sastre et al., 2024b). We analyzed whether the D/E polymorphism differentially altered the association between the channel and the regulatory subunit. CoIP (Fig. 3, A and B) and membrane localization (Fig. 3, C–E) studies were performed. In the coIP studies, we used a tagless Kv1.3 inserted in pcDNA3.1 and CFP-tagged KCNE4 (KCNE4-CFP). Kv1.3/KCNE4 coIP analysis revealed no differences among all KCNE4 mutants (Fig. 3, A and B). To assess plasma membrane localization of the channel, we performed flow cytometry experiments (Bourdin et al., 2016). HEK293 cells expressing Kv1.3 with an extracellular HA tag and different variants of KCNE4-CFP were used (Fig. 3 C). The cells were incubated with a primary antibody against HA (Kv1.3) and a secondary antibody conjugated to Cy5 (Fig. 3 D). The presence of both KCNE4 145D and 145E decreased the Cy5 signal in a similar manner, which correlated with lower levels of Kv1.3 at the plasma membrane (Fig. 3 E).

To further support these data, we performed confocal microscopy–based subcellular localization studies, in which we coexpressed Kv1.3-YFP, different KCNE4-CFP variants, and a membrane marker DsRed (Figs. S1 and S2). No differences in the intracellular retention were observed among the different KCNE4 mutants (Fig. S1). As previously described, pixel-by-pixel analysis of images revealed that Kv1.3, in the absence of KCNE4, mainly targeted the plasma membrane (Sole et al., 2009; Sastre et al., 2024b). However, the coexpression of all three KCNE4 variants similarly altered this distribution, retaining Kv1.3 intracellularly (Fig. S2 A). With a triple-colocalization assay (Sastre et al., 2019), MOC revealed that all KCNE4 variants decreased the plasma membrane targeting of Kv1.3 to a similar degree (Fig. S2 B). KCNE4 membrane expression did not differ between different mutants when it was expressed alone (Fig. S1) or in the presence of Kv1.3 (Fig. S2 C). Furthermore, no changes in Kv1.3/KCNE4 colocalization were observed for any KCNE4 variant (Fig. S2 D).

### The polymorphism did not alter the KCNE4-dependent acceleration of slow inactivation of Kv1.3

**Figure 4.**
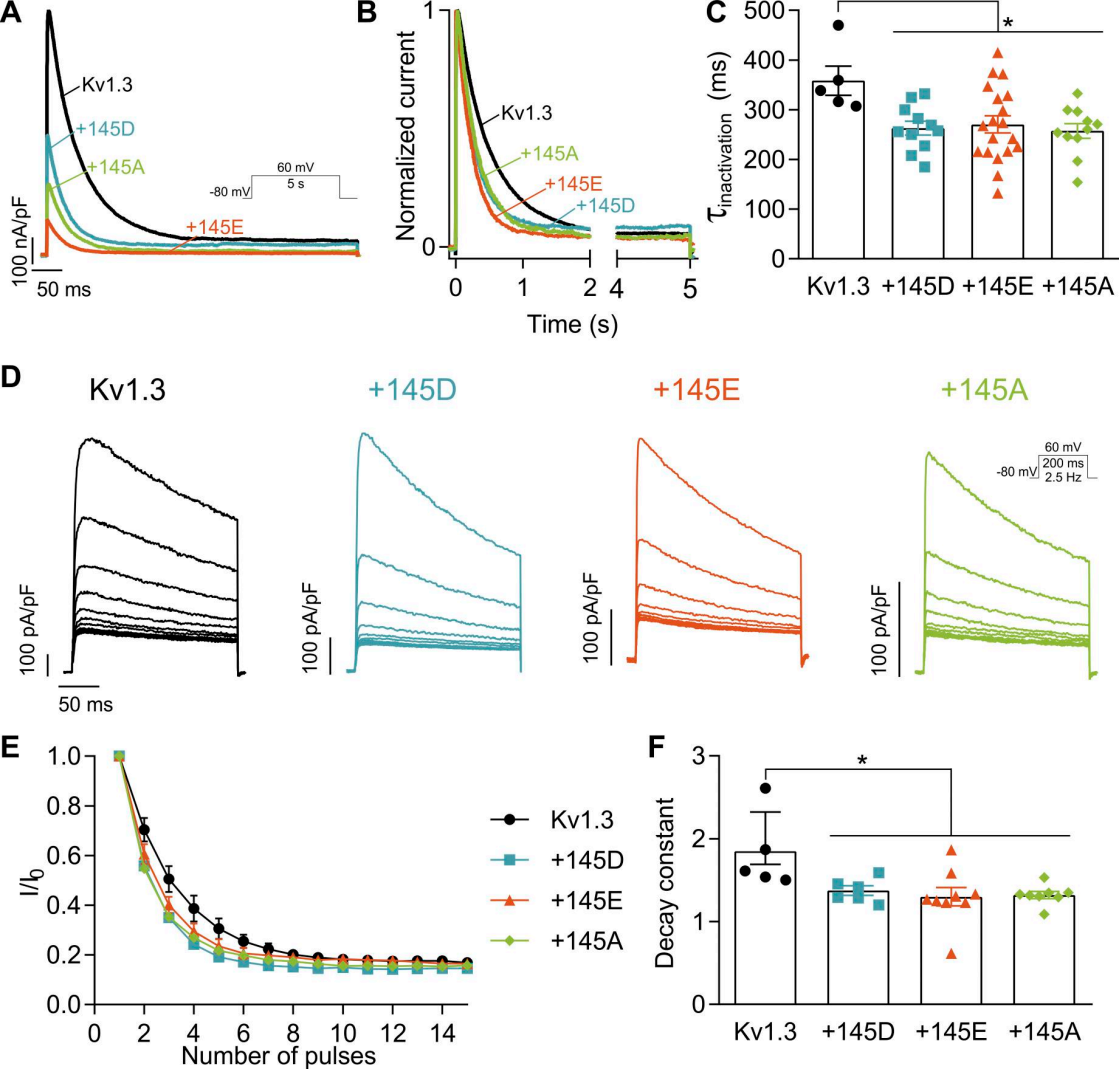
**145D/E polymorphism does not alter the KCNE4-dependent acceleration of slow inactivation of Kv1.3. (A)** Representative 5-s-long traces at +60 mV of HEK293 cells transfected with Kv1.3 without or with KCNE4 (+145D, +145E, +145A). **(B)** Normalization of representative currents in A to visualize the slow inactivation of the channel. **(C)** 5-s traces were fitted with a monoexponential equation to calculate the τ_inactivation_. The values represent the mean ± SE of 5–19 cells for each condition from four different transfections, as depicted. Statistical analysis: one-way ANOVA (P < 0.026) with Tukey’s multiple comparisons test. Specific P values were as follows: *P < 0.05 (0.0344 vs. +145D; 0.0371 vs. +145E; 0.0229 vs. +145A). **(D)** Representative currents of a train of 15 depolarizing pulses at a frequency of 2.5 Hz at +60 mV. **(E)** Relativized peak current for each of the 15 pulses from the protocol in D. **(F)** Current decay from E was fitted to a monoexponential decay to calculate the decay constant. The values represent the mean ± SE of 5–9 cells for each condition, as depicted. In each experiment, two different random conditions—always including Kv1.3 alone—from four different transfections, were recorded. Statistical analysis: one-way ANOVA (P < 0.0108) with Tukey’s multiple comparisons test. Specific P values were as follows: *P < 0.05 (0.0305 vs. +145D; 0.0130 vs. +145E; 0.0188 vs. +145A). Black circles, Kv1.3 alone; blue squares, +145D; orange triangles, +145E; and green diamonds, +145A.

KCNE4 not only promotes Kv1.3 retention in the endoplasmic reticulum (Sole et al., 2009; Sastre et al., 2024b) but also accelerates its characteristic slow C-type inactivation (Sole et al., 2020; Sastre et al., 2024b). Therefore, changes in the C-type inactivation between the variants could reduce the number of available channels, impacting their current density. Hence, we next examined the inactivation of the channel (Fig. 4 and Fig. S3) using long 5-s depolarizing pulses at +60 mV (Fig. 4, A and B). Currents were fitted to monoexponential equations to calculate the τ_inactivation_ (Fig. 4 C). All KCNE4 variants similarly accelerated the inactivation of Kv1.3, and no differences were detected (Table S4). KCNE4 also accelerates the use-dependent or cumulative inactivation of Kv1.3 (Sastre et al., 2024b). Applying a train of 15 depolarizing pulses (Fig. 4 D), the peak current of each pulse was fitted to a monoexponential equation (Fig. 4 E), and the decay constants were calculated (Fig. 4 F and Table S4). Similar to the slow C-type inactivation, all KCNE4s accelerated use-dependent inactivation to a similar degree. Therefore, modulation of channel inactivation is unlikely to be responsible for current density differences between KCNE4 variants.

### Residue charge and position determine KCNE4 polymorphism effects

**Figure 5.**
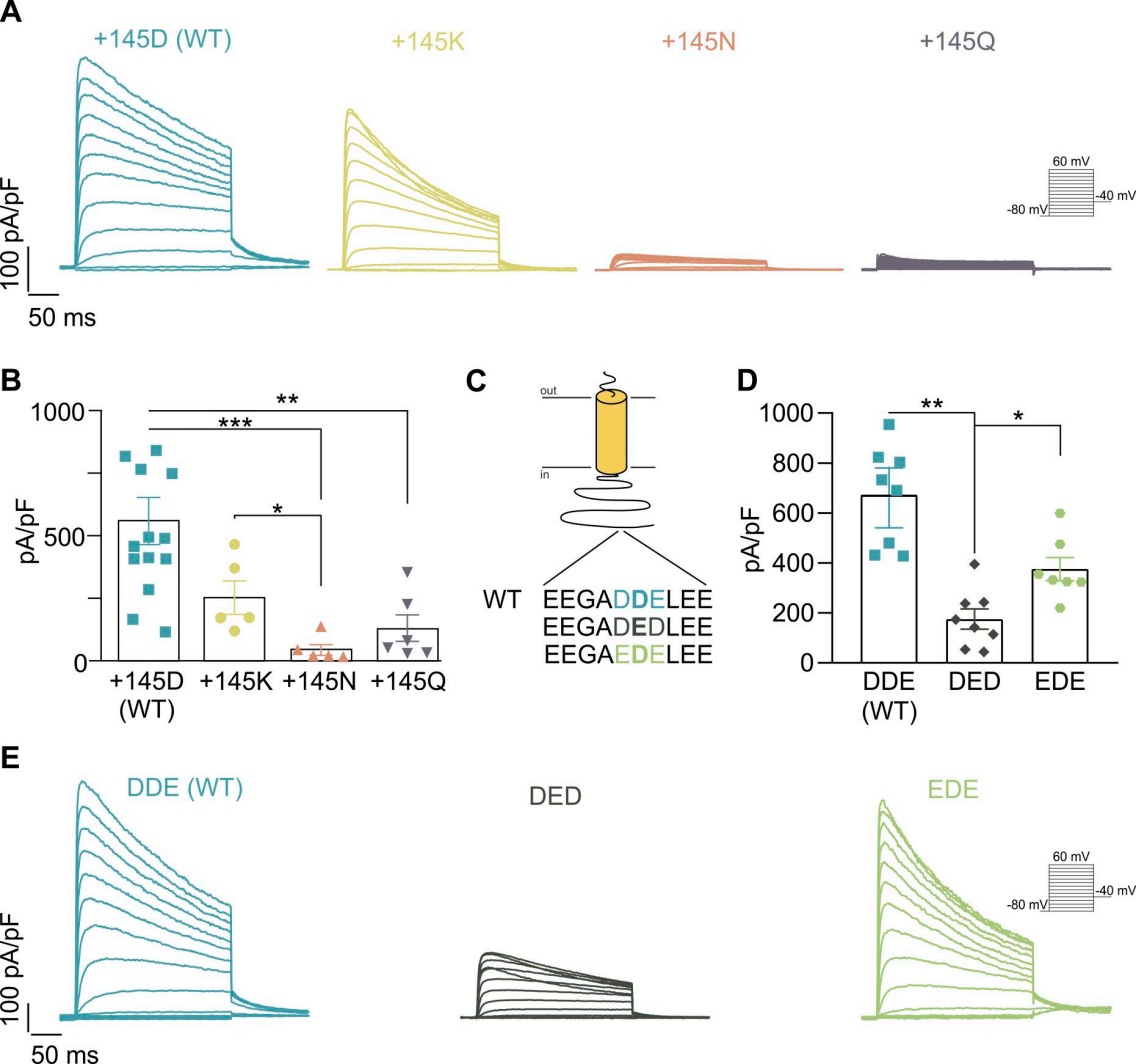
**Charge and position at residue 145 govern KCNE4-dependent Kv1.3 current reduction.** HEK293 cells were transfected with Kv1.3 in the absence or presence of a series of KCNE4 variants. **(A)** Representative currents of Kv1.3 in the presence of KCNE4 WT (145D) and its mutants (+145K, +145N, and +145Q). The cells were held at −80 mV, and the currents were elicited by 250-ms-long pulses to +60 mV in 10-mV steps. Tail currents were measured at −40 mV. **(B)** Peak current density at +60 mV. The values represent the mean ± SE of 5–13 cells from four different transfections, as depicted. Statistical analysis: one-way ANOVA (P < 0.02) with Tukey’s multiple comparisons test. Specific P values were as follows: *P < 0.05 (0.0426 +145K vs. +145N); **P < 0.01 (0.0095 +145D vs. +145Q); ***P < 0.001 (0.0010 +145D vs. +145N). **(C)** Schematic representation of KCNE4 highlighting the anionic cluster containing the distal 145 polymorphic position (in bold). Residues just before and after 145 were mutated to either D or E. **(D)** Peak current density of Kv1.3 in the presence of KCNE4 WT (DDE), KCNE4 DED, and KCNE4 EDE. The values represent the mean ± SE of 7–8 cells from three different transfections, as depicted. Statistical analysis: one-way ANOVA (P < 0.02) with Tukey’s multiple comparisons test. Specific P values were as follows: *P < 0.0292; **P < 0.0025. **(E)** Representative currents from the conditions in panel D using the same protocols as those in panel A.

Although D/E substitutions represent minimal missense mutations, evidence suggests some pathological implications (Shapiro and Koshland, 1994; Brown et al., 2003; Parent et al., 2013; Debler et al., 2016). The KCNE4 145D/E SNP involves either an Asp or a Glu at the polymorphic position, two negatively charged residues whose side chains differ in length by one carbon. Because each variant decreases Kv1.3 currents in different magnitudes, we focused on the properties of the residue. Therefore, the polymorphic position was mutated to Lys (positively charged) and to Asn and Gln (polar uncharged residues). Kv1.3 current density in the presence of KCNE4 145K was within the range of KCNE4 (145D) (Fig. 5, A and B). However, the 145N and 145Q KCNE4 variants significantly decreased Kv1.3 currents.

Interestingly, the SNP is located within a highly anionic cluster of amino acids and is the central residue of a DDE motif (Fig. 5 C). The central Asp (D) is the polymorphic position and is surrounded by another Asp and a Glu. We investigated whether changes in these neighboring Asp and Glu positions also altered KCNE4 modulation of Kv1.3. Thus, we mutated the WT sequence (DDE) to DED and EDE (Fig. 5 C). Interestingly, we determined that solely the central amino acid of the triplet governed the current reduction (Fig. 5, D and E). Thus, unlike the WT (DDE) or alternative EDE, the positioning of an E in the center of the triplet (DED) reduced the Kv1.3 current by twofold over that of KCNE4 WT (Fig. 5 D).

## Discussion

The KCNE families are important ion channel regulators, and mutations are involved in a plethora of diseases (Abbott and Goldstein, 2002). For example, for KCNE4, R20G is linked to atrial fibrillation (Gregers et al., 2017), whereas M58V is associated with thyrotoxic hypokalemic periodic paralysis (Silva et al., 2004). In this work, we investigated the effect of a polymorphism on Kv1.3 located in the distal Ct domain of KCNE4 (rs12621643, 145D/E) that is correlated with immune system alterations such as childhood ALL and allergic rhinitis (Trevino et al., 2009; Freidin et al., 2013). The potassium channel Kv1.3 is involved in the activation and proliferation of leukocytes, and KCNE4, being its ancillary subunit, is a strong modulator of the channel (Conforti et al., 2003; Hu et al., 2007). KCNE4 also regulates Kv7.1 (Ciampa et al., 2011), where the two KCNE4 variants were reported to have antagonistic effects on the channel (Ma et al., 2007); 145D increases Kv7.1 currents, whereas 145E triggers inhibition. However, the participation of the KCNE4 145D/E polymorphism in cardiac pathogenesis is the subject of debate (Hedley et al., 2011; Sinner et al., 2011).

In our findings, we observed differential regulation of the Kv1.3 current amplitude by the 145D/E KCNE4 polymorphism. Unlike on the Kv7.1 channel (Ma et al., 2007), both 145D and 145E produced a significant reduction in the current amplitude in Kv1.3, but the effect was twofold greater with KCNE4 145E. The KCNE4-dependent decrease in the Kv1.3 current has been linked mostly to impairment of forward plasma membrane trafficking of the channel (Sole et al., 2009; Sastre et al., 2024b). However, the presence of KCNE4 variants resulted in no apparent differences in the surface expression of Kv1.3. Because Kv1.3 channels possess a relatively large conductance, a subtle modulation of channel traffic can lead to larger-than-expected effects on the current density.

Although D/E is a minimal missense mutation, important pathogenic consequences have been reported, and differences in methylation claim alterations in protein structure (Shapiro and Koshland, 1994; Brown et al., 2003; Parent et al., 2013; Debler et al., 2016). In addition to KCNE4, natural D/E polymorphisms in other channel proteins have been described. In Kv11.1 (HERG), the E58D variant has a slower inactivation (Anderson et al., 2014), and E637D was identified as a risk variant for long QT syndrome (Amoros et al., 2011). We investigated some variations of the negatively charged residues within the 145-residue–containing triplet. By using the KCNE4 145A mutant, we determined that the presence of an Asp, rather than the absence of a Glu, was responsible for the changes. We also analyzed Kv1.3 currents in the presence of several KCNE4 mutants, such as 145K, 145Q, and 145N, at the polymorphic position. Only the currents in the presence of KCNE4 145K were comparable to those in the presence of WT 145D, whereas for KCNE4 145Q and 145N, the current density was significantly lower. These data suggest that the presence of a charged side chain, independent of its polarity, is necessary to limit how much KCNE4 reduces the current amplitude. The bulk and position of the charge could also impact how this residue interacts with others in KCNE4 or in the Kv1.3–KCNE4 complex. Because the polymorphism is located in a highly intrinsically disordered domain, predicting how this interaction could shape the overall structure of the complex is difficult (Punta et al., 2015; Bondos et al., 2022). In this structure, the polymorphic residue occupies the central position of a negatively charged D–D/E–E motif, and different Asp and Glu mutations within this triplet highlight the importance of the central position for determining the regulatory effect of KCNE4.

Considering the association between the variants and immune system diseases, we still need to examine how this interplay could affect leukocyte physiology. Interestingly, each variant is a risk factor for a different disease. KCNE4 145E is a risk factor for allergic rhinitis (Freidin et al., 2013), whereas 145D is associated with childhood ALL (Trevino et al., 2009). The pathogenesis of allergic rhinitis is mediated by several immune system cell types, including dendritic cells, B cells, and T cells (Zhang et al., 2022), and involves the production of IgE antibodies against inhaled allergens (Bousquet et al., 2020). Further research should explore how lower Kv1.3 activity, a consequence of the KCNE4 145E variant, could participate in this process and in which cell types this effect could be more relevant. On the other hand, in childhood ALL, KCNE4 145D could putatively produce higher Kv1.3 currents. This finding would be consistent with the pathophysiology of chronic lymphoblastic leukemia, in which the overexpression and overactivity of Kv1.3 contribute to disease (Leanza et al., 2013; Szabo et al., 2015), and pharmacological inhibition of Kv1.3 reduces tumor size (Severin et al., 2022). In summary, it is tempting to speculate that this minimal missense mutation, by controlling the Kv1.3 function, could trigger differential pathological outputs.

## Supplementary Material

Table S1 shows statistical analysis.

Table S2 shows voltage-dependent activation constant and τ_inactivation_ of Kv1.3 in the absence (Kv1.3) or presence (+) of KCNE4 variants.

Table S3 shows time constants for the activation and deactivation of Kv1.3 without (Kv1.3) or with (+) polymorphic KCNE4 variants at +60 mV.

Table S4 shows the percentage of inactivation and τ inactivation of Kv1.3 currents after a 250-ms-long pulse at +60 mV and decay constants from a train of 15 depolarizing pulses.

SourceData F3 is the source file for Fig. 3.

## Data Availability

Data are available in the article itself and its supplementary materials.
