# Supplementary material for: Structural determinants at KCNE4 position 145 govern Kv1.3 channel function
Source: J Gen Physiol. 2026 May 20;158(4):e202513936. doi: 10.1085/jgp.202513936 (PMC13189056; doi:10.1085/jgp.202513936)
Supplement: Table S1 — shows statistical analysis. [file jgp_202513936_tables1.docx]

|  | Fig. 2B |
| --- | --- |
| Normality P value (Shapiro-Wilk) | 0.1287 |
| Homoscedasticity (Brown-Forsythe) | 0.0001 |
| Test | Welch's ANOVA test |
| P-value | <0.0001 |
| Post-hoc | Unpaired t with Welch's correction |

|  | Fig. 3B |
| --- | --- |
| Sphericity | 0.310 |
| Significant matching | 0.043 |
| Test | RM one-way ANOVA |
| P-value | 0.310 |
| Post-hoc | - |

|  | Fig. 3E |
| --- | --- |
| Brown-Forsythe ANOVA test | <0,0001 |
| Welch's ANOVA test | <0,0001 |

|  | Fig. 4C |
| --- | --- |
| Normality P value (Shapiro-Wilk) | 0.965 |
| Homoscedasticity (Brown-Forsythe) | 0.212 |
| Test | One-way ANOVA |
| P-value | 0.026 |
| Post-hoc | Tukey’s multiple comparisons |

|  | Fig. 4F |
| --- | --- |
| Normality P value (Shapiro-Wilk) | 0.0697 |
| Homoscedasticity (Brown-Forsythe) | 0.3744 |
| Test | One-way ANOVA |
| P-value | 0.0108 |
| Post-hoc | Tukey’s multiple comparisons |

|  | Fig. 5B |
| --- | --- |
| Normality P value (Shapiro-Wilk) | 0.063 |
| Homoscedasticity (Brown-Forsythe) | 0.405 |
| Test | One-way ANOVA |
| P-value | 0.002 |
| Post-hoc | Tukey’s multiple comparisons |

|  | Fig. 5D |
| --- | --- |
| Normality P value (Shapiro-Wilk) | 0.887 |
| Homoscedasticity (Brown-Forsythe) | 0.865 |
| Test | One-way ANOVA |
| P-value | 0.002 |
| Post-hoc | Tukey’s multiple comparisons |

|  | Fig. S1B |
| --- | --- |
| Normality P value (Shapiro-Wilk) | 0.980 |
| Homoscedasticity (Brown-Forsythe) | 0.096 |
| Test | One-way ANOVA |
| P-value | 0.225 |
| Post-hoc | - |

|  | Fig. S2B |
| --- | --- |
| Normality P value (Shapiro-Wilk) | <0.0001 |
| Homoscedasticity (Brown-Forsythe) | 0.719 |
| Test | Kurskal-Wallis |
| P-value | <0.0001 |
| Post-hoc | Dunn’s multiple comparisons |

|  | Fig. S2C |
| --- | --- |
| Normality P value (Shapiro-Wilk) | 0.001 |
| Homoscedasticity (Brown-Forsythe) | 0.922 |
| Test | Kurskal-Wallis |
| P-value | 0.764 |
| Post-hoc | - |

|  | Fig. S2D |
| --- | --- |
| Normality P value (Shapiro-Wilk) | <0.0001 |
| Homoscedasticity (Brown-Forsythe) | 0.505 |
| Test | Kurskal-Wallis |
| P-value | 0.093 |
| Post-hoc | - |

|  | Fig. S3B |
| --- | --- |
| Normality P value (Shapiro-Wilk) | 0.006 |
| Homoscedasticity (Brown-Forsythe) | 0.002 |
| Test | Kurskal-Wallis |
| P-value | <0.0001 |
| Post-hoc | Dunn’s multiple comparisons |

**Table S1. Statistical analysis.** Summary of the statistical analysis performed with P-values for Shapiro-Wilk normality tests, Brown-Forsythe homoscedasticity test, and the statistical test to compare differences between conditions.
