# Supplementary material for: Structural determinants at KCNE4 position 145 govern Kv1.3 channel function
Source: J Gen Physiol. 2026 May 20;158(4):e202513936. doi: 10.1085/jgp.202513936 (PMC13189056; doi:10.1085/jgp.202513936)
Supplement: Table S2 — shows voltage-dependent activation constant and τinactivation of Kv1.3 in the absence (Kv1.3) or presence (+) of KCNE4 variants. [file jgp_202513936_tables2.docx]

|  | Activation | |  | Inactivation | |
| --- | --- | --- | --- | --- | --- |
|  | V50 | Slope |  | V50 | Slope |
| Kv1.3 | -17.8 ± 0.6 | 5.97 ± 0.49 |  | -27.4 ± 0.4 | 5.23 ± 0.38 |
| +145D | -17.6 ± 0.8 | 7.34 ± 0.73 |  | -27.5 ± 0.2 | 5.16 ± 0.21 |
| +145E | -17.0 ± 0.5 | 6.95 ± 0.45 |  | -23.0 ± 0.6 | 5.38 ± 0.54 |
| +145A | -24.9* ± 0.8 | 5.10 ± 0.63 |  | -32.3 ± 1.1 | 3.95 ± 0.96 |

**Table S2.** Activation and inactivation voltage-dependent constants of Kv1.3 in the absence (Kv1.3) or presence (+) of KCNE4 variants. The values represent mean ± SE of 5–8 independent cells. * p < 0.05 by one-way ANOVA and a Tukey’s *post hoc* multiple-comparison test versus the other conditions.
