# Supplementary material for: Structural determinants at KCNE4 position 145 govern Kv1.3 channel function
Source: J Gen Physiol. 2026 May 20;158(4):e202513936. doi: 10.1085/jgp.202513936 (PMC13189056; doi:10.1085/jgp.202513936)
Supplement: Table S3 — shows time constants for the activation and deactivation of Kv1.3 without (Kv1.3) or with (+) polymorphic KCNE4 variants at +60 mV. [file jgp_202513936_tables3.docx]

|  | τ activation (ms) | τ deactivation (ms) |
| --- | --- | --- |
| Kv1.3 | 1.41 ± 0.14 | 31.2 ± 6.0 |
| +145D | 1.72 ± 0.15 | 31.5 ± 2.3 |
| +145E | 1.56 ± 0.13 | 31.9 ± 3.1 |
| +145A | 1.76 ± 0.19 | 37.3 ± 6.6 |

**Table S3.** Time constants for the activation and deactivation of Kv1.3 without (Kv1.3) or with (+) polymorphic KCNE4 variants at +60 mV. Activation and deactivation processes were fitted to a monoexponential decay to calculate the time constants. The values represent mean ± SE of 5–8 independent cells.
