# Supplementary material for: Structural determinants at KCNE4 position 145 govern Kv1.3 channel function
Source: J Gen Physiol. 2026 May 20;158(4):e202513936. doi: 10.1085/jgp.202513936 (PMC13189056; doi:10.1085/jgp.202513936)
Supplement: Table S4 — shows the percentage of inactivation and τ inactivation of Kv1.3 currents after a 250-ms-long pulse at +60 mV and decay constants from a train of 15 depolarizing pulses. [file jgp_202513936_tables4.docx]

|  | % Inactivation (250 ms) | τ inactivation (ms) | Decay constant |
| --- | --- | --- | --- |
| Kv1.3 | 35.8 ± 3.0 | 358 ± 29 | 2.01 ± 0.32 |
| +145D | 47.0 ± 2.1* | 263 ± 14* | 1.37 ± 0.06* |
| +145E | 50.8 ± 2.1** | 270 ± 17** | 1.30 ± 0.11** |
| +145A | 52.6 ± 2.7** | 257 ± 15** | 1.32 ± 0.04** |

**Table S4.** Percentage of inactivation and τ inactivation of Kv1.3 currents after a 250 ms long pulse at +60 mV and decay constants from a train of 15 depolarizing pulses. The values represent the mean ± SE of 3–19 independent cells. * p < 0.05; ** p < 0.01 by one-way ANOVA and a *post hoc* multiple comparison Tukey test versus control (Kv1.3). No significant differences were observed among the +KCNE4 conditions.
