# Supplementary figures and images for: Structural determinants at KCNE4 position 145 govern Kv1.3 channel function
Source: J Gen Physiol. 2026 May 20;158(4):e202513936. doi: 10.1085/jgp.202513936 (PMC13189056; doi:10.1085/jgp.202513936)

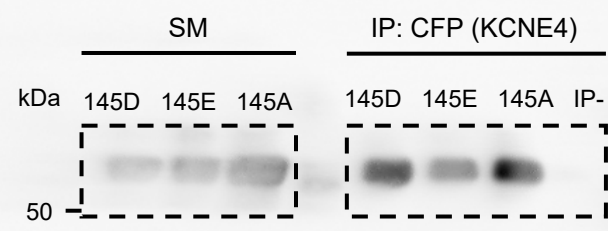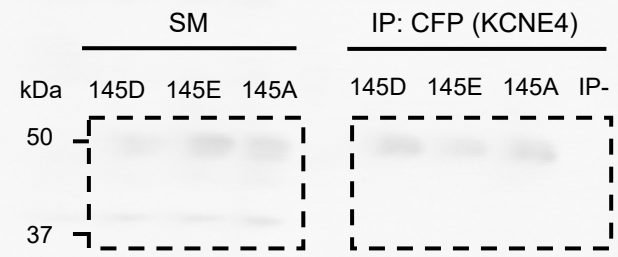

Supplement: SourceData F3 — is the source file for Fig. 3. [file jgp_202513936_sourcedataf3.pdf]
